# Association of IL28B Polymorphisms With the Response to Peginterferon Plus Ribavirin Combined Therapy in Polish Patients Infected With HCV Genotype 1 and 4

**DOI:** 10.5812/hepatmon.13678

**Published:** 2013-11-25

**Authors:** Krzysztof Domagalski, Magorzata Pawlowska, Andrzej Tretyn, Waldemar Halota, Magorzata Tyczyno, Dorota Kozielewicz, Dorota Dybowska

**Affiliations:** 1 Department of Infectious Diseases and Hepatology, Nicolaus Copernicus University, Bydgoszcz, Poland; 2 Department of Plant Physiology and Biotechnology, Nicolaus Copernicus University, Torun, Poland; 3 Centre For Modern Interdisciplinary Technologies, Nicolaus Copernicus University, Torun, Poland

**Keywords:** Hepatitits C, IL28B Protein, Human, Interferon-alpha, Polymorphism, Single Nucleotide

## Abstract

**Background:**

Three single nucleotide polymorphisms (SNPs) near interleukin-28B (IL-28B) gene were shown to be highly associated with treatment response (SVR) in patients with chronic hepatitis C virus (HCV) infection. There is limited data about the role of single and combined IL-28B polymorphisms in HCV-infected Polish population.

**Objectives:**

This study's aim was to determine predictability of three IL-28B gene polymorphisms and other known prognostic factors on the treatment response in HCV genotype 1 and 4 infected Polish patients. The effect of IL-28B polymorphisms on therapy was also compared with other known prognostic factors.

**Patients and Methods:**

We genotyped IL-28B polymorphisms (rs12979860, rs12980275 and rs8099917) by polymerase chain reaction-based restriction fragment length polymorphism assay in a group of 293 patients from which a selected cohort of 174 treatment-naiev patients underwent treatment.

**Results:**

We showed that rs12979860 CC [odds ratio (OR) = 4.6, P < 0.001], rs12980275 AA (OR = 2.9, P = 0.002) and rs8099917 TT (OR = 2.2, P = 0.016) genotypes were associated with successful treatment compared to the rs12979860 CT-TT, rs12980275 AG-GG and rs8099917 TG-GG, respectively. Patients bearing of IL-28B profile including the three favourable genotypes do not have much chance of a recovery (OR = 3.4, P = 0.002). Except for IL-28B polymorphisms, there was no association of SVR with any other pretreatment clinical data in analyzed group. The correlation of SNPs with other host and viral factors revealed association of favorable genotypes of IL-28B markers with high levels of alanine aminotransferase and baseline HCV viral load.

**Conclusions:**

IL-28B polymorphisms were the strongest pretreatment predictors of response to pegylated interferon and ribavirin in Polish patients chronically infected with HCV genotype 1 and 4. This study confirm the strongest impact of IL-28B rs12979860 on SVR, nevertheless rs12980275 AA seems to be more important than rs8099917 TT in predicting positive treatment response.

## 1. Background

Hepatitis C virus (HCV) infection is a major health problem worldwide (1). The overall worldwide prevalence of HCV varies geographically and in European countries ranges from 0.5% to over 3% (2). In Poland the rate is 1.9% which means circa 730 thousand infected people (3). Despite the optimization of existing treatment programs, the current effectiveness of treatment of chronic hepatitis C with pegylated interferon alpha (peg-IFNα) and ribavirin (RBV) in patients infected with difficult-to-treat HCV genotypes, such as types 1 and 4, commonly occurring in Poland, is unsatisfactory (4). In addition to the limited efficacy of the treatment, this therapy involves a lot of adverse side effects of varying types and intensity (5). Many prognostic factors affecting the final treatment outcome in hepatitis C have been discovered and studied (6, 7). Therefore, identifying predictors of response to current therapy, particularly in patients infected with genotype 1 and 4, remains as one of the main objectives of the research. It is still unknown why patients characterized by similar clinical parameters, differ in response to treatment, hence the need to seek new independent predictors of the efficacy of genetically-based therapy connected with the human organism, in relation to particular HCV genotypes and current treatment program.

Recently, genome-wide association study (GWAS) has independently demonstrated that single nucleotide polymorphisms (SNPs) close to interleukin 28B gene (IL-28B), are strongly associated with HCV patients’ response to peg-IFNα and RBV treatment (8-11) and spontaneous elimination of the virus (10, 12) in different populations. Thus, IL-28B variants may represent host factors determining susceptibility to HCV treatment, which may provide a genetic explanation for the different outcomes of HCV infection in various hosts. Among detected SNPs, the most important ones seem to be rs12979860 (C/T), rs8099917 (T/G), and rs12980275 (A/G).

## 2. Objectives

The relationship between the response to treatment and the single and combined profiles three most popular IL-28B genetic determinants (rs12979860, rs12980275 and rs8099917) in HCV-infected Polish population has not been previously evaluated. In the presented study, we showed the role of polymorphisms near the IL-28B gene in predicting the outcome of pegylated interferon/ribavirin combined therapy in Polish patients, infected with HCV genotypes 1 and 4.

## 3. Patients and Methods

### 3.1. Patients and Clinical Data

According to inclusion criteria (chronic 1 or 4 HCV infection) and according to exclusion criteria (hepatitis B or human immunodeficiency virus (HIV) co-infection, coexistent autoimmune liver diseases, hemochromatosis, or other coexistent chronic liver diseases) overall, 298 chronic HCV adult patients of Caucasian ethnicity, from the region of Central Poland, infected with genotypes 1 or 4 HCV, were included in the study. This experimental study was approved by the Ethics Committee of Collegium Medicum Nicolaus Copernicus University, Bydgoszcz, Poland. Written informed consent was obtained from each participant. From this group we retrospectively selected a cohort of 174 naive adult patients, who were routinely treated with combined antiviral therapy with peg-IFNα 2a or 2b and RBV between the years 2008 and 2012 in one of the Polish academic centers. Patients underwent treatment under a standard of care (SOC) protocol with peginterferon (Pegasys, PegIntron) and weight-based ribavirin for 48 weeks.

In assessing the effectiveness of antiviral treatment, virological response criteria were used. HCV RNA levels were determined by quantitative PCR at baseline, week 12, and by qualitative PCR at the end of treatment, and 24 weeks after the end of therapy. To quantitative PCR assay we using the COBAS AmpliPrep/COBAS TaqMan HCV RNA Test (Roche Molecular Diagnostics). The primary endpoint of the study was sustained virological response (SVR), defined as undetectable HCV RNA in serum 24 weeks after the completion of therapy. The secondary endpoint was early virological response (EVR) defined as a reduction of HCV RNA by at least 2 logs after the first 12 weeks of treatment compared with baseline HCV RNA levels. Patients who failed to achieve early virological response (EVR) were considered to be non-responders, and their therapy was discontinued. Also, non-responder was defined as patient with detectable HCV RNA in serum at any other time during the 48 week therapy. EVR with undetectable HCV RNA in serum was referred as a complete EVR (cEVR).

### 3.2. DNA Extraction and IL-28B Genotyping

In order to carry out the genotyping analysis, genomic DNA was prepared by extraction method using Igepal CA-630 detergent from peripheral blood samples collected in 0.5 M EDTA tubes (13). Based on the published human chromosome 19q13 sequence (NCBI Reference Sequence: NT_011109.16) the genotyping of SNPs rs12979860, rs12980275, and rs8099917 was carried out by polymerase chain reaction (PCR), and restriction fragment length polymorphism (RFLP), as described previously (14). PCR products from 30 samples from the same patient group were further sequenced to confirm the PCR-RFLP genotyping results.

### 3.3. Statistical Analyses

To identify factors predicting treatment response, we evaluated the statistical significance in univariate analysis of the relationships between clinical characteristics and the response phenotype. Continuous variables like HCV viral load and ALT activity were compared with Mann-Whitney *U* tests. The Pearson chi-square or Fisher's exact test, where appropriate, were used for categorical variables. IL28B SNP comparisons were made using a dominant model, in which patients carrying one or two copies of minor allele were compared with others. For all these tests, two-tailed p-values were used and P values < 0.05 were considered to be statistically significant. Odds ratios (ORs) and 95% confidence intervals (95% CIs) were also calculated for association test between the IL-28B polymorphisms and the binary clinical variables. Statistical analysis was performed with the SPSS software version 20 for Windows.

## 4. Results

### 4.1. Patient Characteristics Associated With SVR 

The demographic and clinical characteristics of the treated patients in whole group, stratified by SVR, are presented in Table 1. According with response to therapy, 59 patients obtained SVR (33.9%), 71 patients were non-responders (40.8%) and 44 patients were relapsers (25.3 %). HCV viral load decline more than 2 logs at week 12 during therapy (EVR) affected 143 (81.6%) of patients. Among 143 patients who achieved EVR, 59 (41.5%) achieved SVR, but highest SVR rate was achieved in patients with cEVR (41/73, 56.2%). As shown in Table 1, there were no statistically significant differences in the baseline characteristics between SVR and no SVR groups. In selected subgroups for genotype 1 or 4 HCV, treatment efficacy was 34.8% and 30.3% respectively and the observed difference was not statistically significant. 

**Table 1.** Characteristics of Chronic HCV Patients Treated With Peg-IFN and RBV According to Final Treatment Response

Characteristic	All Patients (n = 174)	SVR, 59 (33.9%)	R/NR, 115 (66.1%)	P value, SVR vs R/NR
**Age at the end of treatment, y**				
Median (range)	34 (18-74)	34 (19-70)	34 (18-74)	0.535
> 45, No. (%)	52 (29.9)	17 (28.8)	35 (30.4)	0.825
**Gender, No. (%)**				
Women	78 (44.8)	32 (54.2)	46 (40.0)	0.074
Men	96 (54.2)	27 (45.8)	69 (60.0)	0.074
**BMI, No. (%)**				
> 25 kg/m^2^	37 (21.3)	11 (18.6)	26 (22.6)	0.545
**Liver fibrosis (F) stage (Scheuer ), No. (%)** ^**a**^				
Available	153 (86.4)	49 (83.1)	104 (90.4)	
F0-F1	98 (64.1)	21 (42.9)	70 (67.3)	0.221
F2-F4	55 (35.9)	28 (57.1)	34 (32.7)	0.221
**Liver activity (A) grade (Scheuer), No. (%)**				
Available	153	49 (83.1)	104 (90.4)	
A0-A1	40 (26.1)	12 (24.5%)	28 (26.9)	0.749
A2-A4	113 (73.9)	37 (75.5)	76 (73.1)	0.749
**Baseline ALT , U/L** ^**a**^				
median (range)	52 (10-608)	48 (10-299)	52 (12-608)	0.269
> 40 U/l	109 (62.6)	34 (57.6)	75 (65.2)	0.327
**Baseline Viral Load, IU/ml**				
median (range), 10^5^	5.66 (0.037-89.2)	5.13 (0.04-71.9)	6,52 (0.20-89.2)	0.224
≥ 600000	85 (48.9)	27 (45.8)	58 (50.4)	0.559
≥ 800000	72 (41.3)	21 (35.6)	51 (44.3)	0.267
**HCV genotype, No. (%)**				
1	141 (81.0)	49 (83.1)	92 (80.0)	0.741
4	33 (19.0)	10 (16.9)	23 (20.0)	0.741
**12 weeks of treatment, No. (%)**				
EVR ^a^	142 (81.6)	59 (100.0)	83 (72.2)	- ^b^
cEVR ^a^	73 (42.0)	41 (69.5)	32 (27.8)	- ^b^
**Final outcome, No. (%)**				
SVR ^a^	59 (33.9)			
R ^a^	44 (25.3)			
NR ^a^	71 (40.8)			

^a^ Abbreviations: ALT, alanine aminotranferase; SVR, sustained viral response; EVR, early viral response; cEVR, complete EVR; R, relapse; NR, no response; Scheuer, modified Scheuer scoring system

^b^ not tested due to discontinuation of treatment in patients who did not achieve EVR

### 4.2. IL-28B Polymorphisms Genotype Frequencies, Covariation and Haplotypes

In 293 patients group, the analysis of the incidence of genotypes for IL-28B SNPs was carried out. Genotype distributions at all 3 SNPs were in accordance with Hardy-Weinberg equilibrium. The genotypic frequency of rs12979860 CC was 27.1%, CT was 56.4%, and TT was 16.5%. The proportion for rs8099917 TT was 50.3%, TG 43.8% and GG 5.9%, and for rs12980275 AA was 30.2%, AG 53.5% and GG 17.3%. The Linkage disequilibrium (LD) analysis show that these SNPs were in strong LD, but not complete; for the most distanced from each other on the chromosome rs12979860 and rs12980275 (D’ = 0.902, r^2^ = 0.786), and for rs12979860 and rs8099917 (D’ = 0.999, r^2^ = 0.635), as well as for rs8099917 and rs12980275 (D’ = 0.999, r^2^ = 0.658). Haplotypes consisting of 3 alleles from rs12979860, rs8099917 and rs12980275 such as CTA, TGG and TTG appeared most frequently (50%, 29% and 15% respectively). At the same time, CGA and CGG haplotypes was absent. All patients with rs12979860 CC had also rs8099917 TT and in most cases showed a lack of rs12980275 GG. On the other hand, the presence of rs8099917 TT was connected with the occurrence of not only rs12979860 CC but also with rs12979860 CT and TT.

### 4.3. Association of IL-28B Polymorphisms With Treatment Response

As previously shown (Table 1), bio-clinical characteristics are not related to the type of the final response to therapy in the analyzed group of patients undergoing treatment. In contrast, a significant relationship was observed between response type and IL-28B SNP genotype (Table 2). The dominant genotype distributions for IL-28B rs12979860 (CC vs CT and TT), rs12980275 (AA vs AG and GG) and rs8099917 (TT vs TG and GG) polymorphisms were significantly different between SVR group and no SVR grup (P < 0.001, P = 0.002 and P = 0.016, respectively). The strongest prediction was shown for IL-28B rs12979860. In the analyzed group of patients for rs12979860, the odds ratio (OR) of being a responder for CC genotype as compared to genotypes CT and TT was 4.6 (95% CI = 2.2-9.7). The SVR was achieved in 61.5% of patients with the genotype CC of rs12979860, compared with 26.7% in patients with the genotype CT, and 23.3% in patients with the genotype TT. 

**Table 2.** Role of IL-28B Gene Polymorphisms on Virologic Response in Patients Infected With HCV Genotypes 1 and 4

	Treatment Response			
	**EVR ** ^a^	**cEVR ** ^a^	**ETR **	**SVR ** ^a^
**IL-28B SNP**				
CC rs12979860 (n = 39)	38 (97.4)	28 (71.8)	45 (33.3)	24 (61.5)
CT-TT rs12979860 (n = 135)	105 (77.8)	33 (84.6)	62 (45.9)	28 (26.7)
OR ^a^ (95% CI ^a^)	10.8 (1.4-83.3)	5.1 (2.3-11.1)	6.4 (2.5-16.5)	4.6 (2.2-9.7)
P value	0.004	< 0.001	< 0.001	< 0.001
**IL-28B SNP**				
TT rs8099917 (n = 87)	76 (87.4)	44 (50.6)	29 (29.0)	37 (42.5)
TG-GG rs8099917 (n = 100)	67 (67.0)	57 (65.5)	38 (38.0)	22 (22.0)
OR (95% CI)	2.1 (0.9-4.6)	2.0 (1.1-3.8)	2.4 (1.3-4.5)	2.2 (1.2-4.2)
P value	0.075	0.021	0.004	0.016
**IL-28B SNP**				
AA rs12980275 (n = 46)	44 (95.7)	31 (67.4)	35 (76.1)	24 (52.2)
AG-GG rs12980275 (n = 128)	99 (77.3)	42 (32.8)	60 (46.9)	35 (27.3)
OR (95% CI)	6.5 (1.5-28.6)	4.2 (2.1-8.7)	3.6 (1.7-7.7)	2.9 (1.4-5.8)
P value	0.006	< 0.001	0.001	0.002

^a^ Abbreviations: CI, confidence interval; OR, odds ratio; SVR, sustained viral response; ETR, end of treatment response; EVR, early viral response; cEVR, complete EVR

Analyses of the combined connection of examined genotypes for SVR results showed that patients bearing these three favorable marker genotypes do not have much chance of a recovery (CC/TT/AA vs the other, OR = 3.4, 95% CI = 1.6-7.3, P = 0.002) when compared to patients whose only marker taken into account is rs12979860. Also, the presence of profiles comprising at least one of the favourable genotypes did not yield significant increase in SVR rates (CC or TT or AA vs other, OR = 2.19, 95% CI = 1.15- 4.15, P = 0.016). Analysis of the relationship between haplotypes and SVR showed statistically significant differences between the most common beneficial favourable CTA haplotype, and adverse TGG haplotype (OR = 2.19, 95% CI = 1.12 - 4.28), P < 0.05). A significant relationship was observed between EVR, cEVR, ETR and IL-28B SNP genotype (Table 2). The distribution of genotypes CC and CT-TT rs12979860 most significantly divided the patients in terms of the frequency of achieving EVR (OR = 10.8, P < 0.001), cEVR (OR = 5.1, P < 0.001) and ETR (OR = 6.4, P < 0.001). In a group of 39 people with CC rs12979860 genotype, only one patient did not achieve EVR. No significant difference was found only between TT/TG-GG genotypes of rs809917 and EVR results. In the group of 73 people who achieved cEVR for all analyzed markers there were no statistically significant differences in the distribution of genotypes among subgroups of patients with a different SVR result (Table 3). The chances for SVR in patients achieving EVR are differentiated only by markers rs12979860 and rs12980275. 

**Table 3.** Impact of IL28B SNP on SVR in Patients Who Achieved EVR and cEVR

	IL28B SNP		
	rs12979860	rs8099917	rs12980275
**Genotype**	CC/CT-TT	TT/TG-GG	AA/AG-GG
**Response, EVR group, (SVR rates, %)**	24/35 (63.2/33.3)	37/22 (48.7/32.8)	24/35 (54.5/35.4)
OR (95% CI)	3.4 (1.5-7.4)	1.9 (0.9-3.8)	2.2 (1.1-4.5)
P value	0.001	0.055	0.031
**Response, cEVR group (SVR rates, %)**	18/23 (64.3/51.1)	28/13 (63.6/44.8)	19/22 (61.3/52.4)
OR (95% CI)	1.7 (0.6-4.5)	2.2 ( 0.8-5.6)	1.4 (0.6 -3.7)
P value	0.270	0.113	0.448

### 4.4. Association of IL-28B Polymorphisms With Baseline Viral Load and ALT Activity

**Figure 1.**
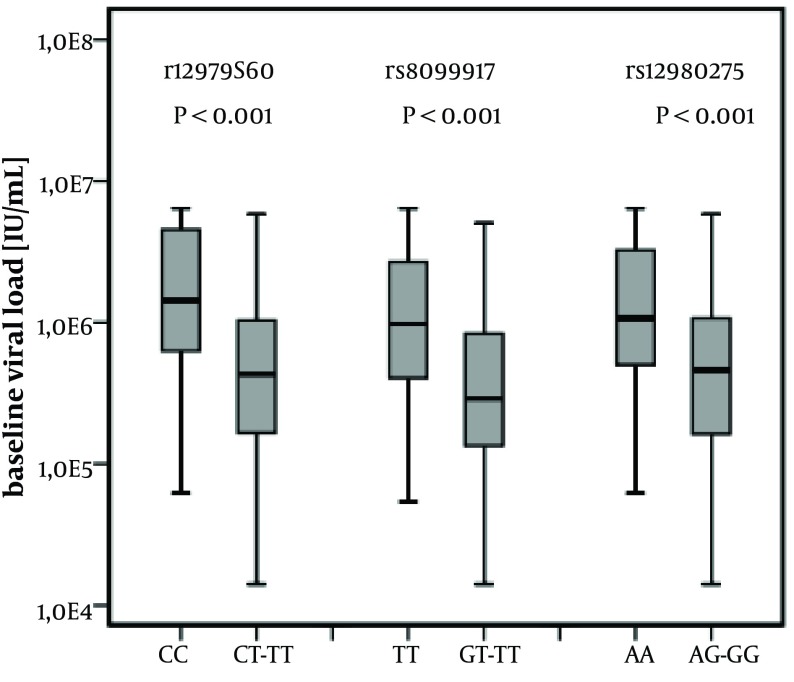
The Association of IL-28B SNP With Baseline Viral Load in Chronic HCV Patients There were significant differences in viral load between patients carrying CC and CT-TT rs12979860, TT and TG-GG rs8099917 and AA and AG-GG rs12980275 genotypes. Data are median with 10th, 25th, 75th and 90th percentiles as vertical boxes with error bars.

Despite there being no significant differences between baseline viral load and final treatment response among the analyzed groups, a highly significant relationship between baseline viral load and all analyzed IL-28B markers was shown (Figure 1). Median baseline viral load for patients with genotype CC of rs12979860 was 14.3 × 10 ^5 ^and for patients with genotype CT-TT was 4.3 × 10 ^5 ^(P < 0.001). Analysis for rs12980275 and rs8099917 revealed similar results. Baseline viral load was higher in patients with either AA rs12980275 (10.7 × 10 ^5 ^), or TT rs8099917 (9.7 × 10 ^5 ^), genotype, compared with patients with AG-GG rs12980275 (4.6 × 10 ^5 ^) or TG-GG rs8099917 (2.9 × 10^5^) genotypes, respectively, and these differences were highly significant (P < 0.001). Moreover, the baseline viral load was analyzed as a categorical variable using known cut-off level of 600 000 IU/mL or 800 000 IU/mL (Table 4). For a cut-off of 600 000 IU/mL and 800 000 IU/mL we showed that favorable genotypes of all analyzed markers were associated with high viral load. 

**Table 4.** Impact of IL-28B SNP on High Baseline Viral Load According to 600000 and 800000 IU/mL Stratification

	Baseline Viral Load, IU/mL	
	≥ 600000	≥ 800000
**IL-28B SNP**		
CC rs12979860	30 (76.9)	26 (66.6)
CT-TT rs12979860	55 (40.7)	46 (34.1)
OR (95% CI)	4.8 (2.1-11.0)	3.9 (1.8-8.2)
P value	< 0.001	< 0.001
**IL-28B SNP**		
TT rs8099917	57 (65.5)	49 (56.3)
TG-GG rs8099917	28 (32.2)	23 (26.4)
OR (95% CI)	4.0 (2.1-7.5)	3.5 (1.9-6.8)
P value	< 0.001	< 0.001
**IL-28B SNP**		
AA rs12980275	32 (69.6)	29 (63.0)
AG-GG rs12980275	53 (41.4)	43 (33.6)
OR (95% CI)	3.2 (1.5-6.6)	3.4 (1.7-6.8)
P value	0.001	0.001

**Figure 2.**
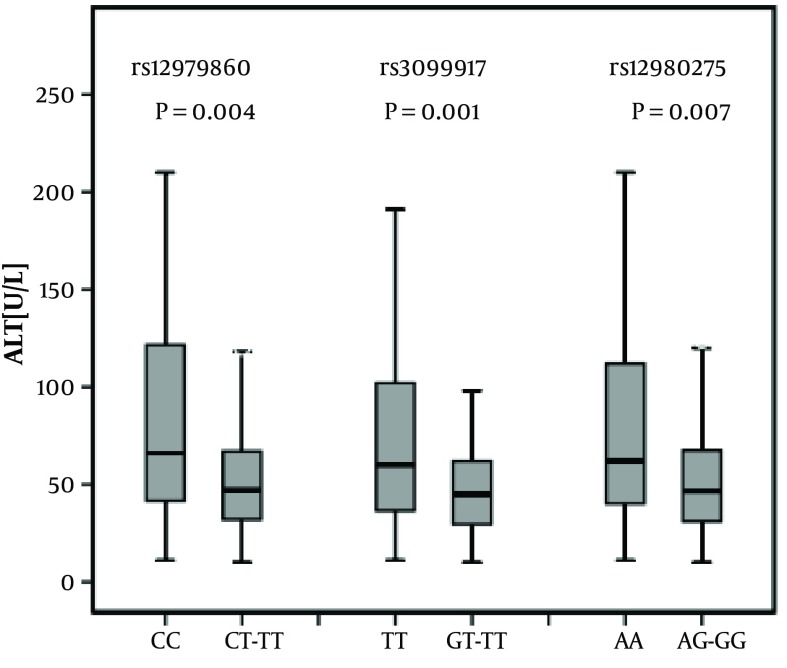
The Association of IL-28B SNP With Baseline ALT Activity in Chronic HCV Patients The ALT levels were significantly lower in patients carrying the unfavorable alleles (T of rs12979860, G of rs8099917 and G of rs12980275). Data are median with 10th, 25th, 75th and 90th percentiles as vertical boxes with error bars.

The existence of statistically significant differences in ALT activity between favorable and unfavorable genotypes of each of the analyzed markers of IL-28B was observed (Figure 2). The presence of favorable genotypes for all three IL-28B markers was connected with an increase in ALT activity. The largest differences were observed for the rs12979860 marker. 

## 5. Discussion

In this retrospective study we validated the clinical significance of the IL-28B polymorphisms on treatment outcome using relatively large Polish mono-centric cohort patients chronically infected with a difficult to treat HCV genotypes 1 and 4, and compared its significance with other host factors. The genotype frequency of analyzed markers is variable across different ethnic groups and among patients infected with different, in terms of difficulty of treatment, HCV genotypes (10, 11, 15). Interestingly, our results by using a relatively large cohort indicated a decreased percentage of favorable genotypes, such as CC rs12979860, rs8099917 TT and rs12980275 AA, compared to Caucasian patients groups with chronic hepatitis C (10, 11, 16, 17). While in the European studies of the Caucasian race nearly 40% incidence of the CC genotype of rs12979860 is observed, the incidence rate for patients in the study population did not exceed 30%. In two independent studies performed on 142 and 97 Polish patients infected with genotype 1 HCV, distribution of rs12979860 CC genotype was 26.8% and 21%, respectively (18, 19). Additionally, the reduced frequency of favorable genotypes rs12979860 CC, rs8099917 TT and rs12980275 AA was showed in our study, when we examined children and adolescents infected with HCV genotype 1 and 4 (14). The analysis of predictive value of IL-28B SNP showed that these markers significantly affect the chances of success of combined therapy. Among the discussed markers, the most predictive one is rs12979860. The other two markers (rs8099917, rs12980275) were also connected with the response to therapy, but their predictive value was lower. These results confirm previous analyzes conducted on Caucasian patients exhibiting predictive characteristics of IL-28B markers (9, 11, 16, 20, 21). In our study the existence of linkage disequilibrium was shown between the analyzed markers, and thanks to direct analysis of the total impact of discussed markers on the prediction of antiviral response it has been established that these genetic markers do not accumulate their predictive properties. Comparison of the obtained data with the results of the analysis of genotypes for the strongest marker indicates less predictive capacity for the preferred haplotype CTA and for patients with three favorable genotypes of analyzed markers. Hence the analysis of the connection between host’s genetic variability and the result of therapy can be based on examining the strongest one of the discussed markers. In the coming years we will be able to use in the treatment of HCV, in addition to combined therapy, triple therapy characterized by increased efficiency, especially in patients with unfavorable IL-28B genotypes markers (22). Also we found a connection between IL-28B polymorphisms and SVR in cohort patients who achieved EVR and no impact in patients with cEVR. It has yet to be determined whether the combination of IL-28B SNPs genotype and EVR can be used to optimize SVR rates (23, 24). We also demonstrated that the IL-28B rs12979860 and rs129780275 polymorphisms affect the EVR. However, all three markers have a statistically significant influence on cEVR and ETR. These observations confirm associations of IL-28B markers with viral eradication on treatment in HCV1 and 4 genotype infection (17, 25, 26). A study by Mach et al. performed on Polish patients with chronic HCV genotype 1, demonstrated that rs12979860 polymorphism was the predictor of EVR, ETR and SVR, which is a finding similar to ours (18). In a recent study, the independent role of rs12979860 in SVR among Polish patients with HCV mono and HIV/HCV co-infection, was found (27). Nevertheless, these studies did not analyze rs8099917 and rs12980275. Additionally, contrary to Mach et al. results, we showed significant association of IL-28B rs12979860 CC genotype (as well as rs8099917 TT and rs12979860 AA) with higher baseline viral load and higher levels of ALT. Also, the correlation between IL-28B genotypes and baseline viral loads was not demonstrated in Jablonowska et al. study. However, these associations were shown in studies conducted on Caucasian patients (11, 16). Lindh et al. presented model explaining the correlations between IL-28B-related genotypes and viral RNA levels. The model presumes that spontaneous resolution is more common in patients with lower viral loads and carrying favourable genotype, and therefore the HCV RNA levels in chronic infection become higher in patients carrying the favourable genotypes than in those with the unfavourable genotypes (28). In our study we determined that baseline characteristics do not significantly correlate with SVR rate in combined therapy. Nonetheless, a study including Polish patient group with HCV (genotypes 1, 3, and 4) mono-infection and HIV/HCV co-infection, showed that the age >40 years independently influences SVR (27). In our study performed on children and adolescents infected with difficult-to-treat HCV genotypes 1 and 4, we showed that except IL-28B polymorphisms, there was no association of SVR with any other clinical data, which is a result similar to the one obtained in adult patients studies (14). The lack of statistically significant differences in bio-clinical characteristics does not contradict earlier reports, and indicate that among the known predictors of therapy outcome, the discussed genetic markers within the IL-28B are the most important ones in the prediction of the final therapeutic effect.
